# Maternal morbidity in the first year after childbirth in Mombasa Kenya; a needs assessment

**DOI:** 10.1186/1471-2393-9-51

**Published:** 2009-11-05

**Authors:** Matthew F Chersich, Nicole Kley, Stanley MF Luchters, Carol Njeru, Elodie Yard, Mary J Othigo, Marleen Temmerman

**Affiliations:** 1 Department of Obstetrics and Gynaecology, Ghent University, Belgium; 2 International Centre for Reproductive Health, Mombasa, Kenya; 3 Reproductive Health and HIV Research Unit, University of Witwatersrand, South Africa; 4 Coast Provincial General Hospital, Mombasa, Kenya

## Abstract

**Background:**

In sub-Saharan Africa, few services specifically address the needs of women in the first year after childbirth. By assessing the health status of women in this period, key interventions to improve maternal health could be identified. There is an underutilised opportunity to include these interventions within the package of services provided for woman-child pairs attending child-health clinics.

**Methods:**

This needs assessment entailed a cross-sectional survey with 500 women attending a child-health clinic at the provincial hospital in Mombasa, Kenya. A structured questionnaire, clinical examination, and collection of blood, urine, cervical swabs and Pap smear were done. Women's health care needs were compared between the early (four weeks to two months after childbirth), middle (two to six months) and late periods (six to twelve months) since childbirth.

**Results:**

More than one third of women had an unmet need for contraception (39%, 187/475). Compared with other time intervals, women in the late period had more general health symptoms such as abdominal pain, fever and depression, but fewer urinary or breast problems. Over 50% of women in each period had anaemia (Hb <11 g/l; 265/489), with even higher levels of anaemia in those who had a caesarean section or had not received iron supplementation during pregnancy. Bacterial vaginosis was present in 32% (141/447) of women, while 1% (5/495) had syphilis, 8% (35/454) *Trichomonas vaginalis *and 11% (54/496) HIV infection.

**Conclusion:**

Throughout the first year after childbirth, women had high levels of morbidity. Interface with health workers at child health clinics should be used for treatment of anaemia, screening and treatment of reproductive tract infections, and provision of family planning counselling and contraception. Providing these services during visits to child health clinics, which have high coverage both early and late in the year after childbirth, could make an important contribution towards improving women's health.

## Background

In recent years, maternal health services in resource-constrained settings have increasingly focused on the importance of skilled birth attendants and the management of intrapartum complications [1]. Also, much efforts have been made to rationalise the package of services for antenatal care [2,3]. Antenatal care coverage remains high in most of Africa and the proportion of births which occur within medical services is steadily increasing [4,5]. By contrast however, few resources are allocated to delivering specific services for women in the year after childbirth. The essential package of postpartum services is poorly defined, and the optimum structure and number of visits for these services remains uncertain [6]. Given weaknesses in the current model for health services for women after childbirth, it is worthwhile to examine alternative configurations of service delivery. Most notably, contact with health services in the year after childbirth presents a vital opportunity to assist women return to full health, and to prevent sexual and reproductive health problems in this period.

The postpartum period, defined by WHO as beginning one hour after delivery of the placenta and continuing until six weeks after childbirth [7], is a period of substantial transition as women move from pregnancy and childbirth back to reproductive potential. Many studies in Africa refer to the postpartum period in more general terms, even including up to two years after childbirth [8-12]. Some definitions of maternal mortality include late maternal deaths (deaths between six weeks and one year after childbirth) [13]. This acknowledges the fact that some physiological effects of pregnancy still occur in this period and that the health of women at this time warrants specific attention [14,15]. The pre-pregnant state has not yet completely returned within six weeks of childbirth, lactation usually continues throughout the first year, often the menstrual cycle has not yet normalized and sexual activity may not have resumed. Further, with screening, detection and treatment of diseases at several time points in the year after giving birth, the safety of future pregnancies could be improved, as well as offering the inherent benefits of improvements in women's overall health status.

Though in many settings over half of maternal deaths occur after childbirth [16,17], data on the health of women during this time are sparse. Moreover, reports usually focus on life-treating obstetric complications such as postpartum haemorrhage with a relative neglect of important common morbidities [7,18-20]. Thus, while several previous studies have highlighted the contribution of the postpartum period to maternal mortality, few have described the burden of more common chronic conditions such as anaemia and depression [21-23]. In addition, most recent studies among postpartum women in Kenya have concentrated on HIV-infected women [8,10,11], and additional information is required about the burden of disease in women without HIV infection, who constitute the majority in these settings.

This study describes acute and chronic morbidity in the year after childbirth, which can be considered as either: conditions associated with pregnancy and childbirth; diseases specific to the postpartum period (such as those related to breastfeeding or the return of reproductive potential); or as intercurrent illnesses (such as cervical neoplasia). Further, the study sets out how patterns of sexual behaviour and morbidity vary with the length of time since delivery. Identifying the needs of women in the year after childbirth would inform selection of essential interventions to secure the health of women during this period, tailored to their changing needs over this time course. Provision of these interventions for women bringing their child for health care could achieve high population coverage as child-health services have good uptake in most countries [24].

## Methods

At Coast Provincial General Hospital in Mombasa, Kenya, 500 women attending an immunization and acute care paediatric clinic participated in a cross-sectional survey to describe the levels of maternal morbidity in the year after childbirth. The survey, held in 2006, also investigated the feasibility of providing HIV testing and counselling, and the levels of HIV-related disease in this population [25,26]. Study activities formed part of a project to improve maternal health services in Coast Province Kenya. From 2002 to 2005 this project focused on strengthening antenatal and intrapartum services; however as it appeared that few women attended postpartum services and their needs were ill defined, this survey was planned to assess women's needs after childbirth and to define a service package that could be provided for women bringing their child for health care. The study was approved by the Kenyatta National Hospital Ethics and Research Committee.

The study population consisted of consecutive women who were older than 16 years, biological mothers of the child, and between four weeks and one year after childbirth.

After completion of the child-health visit, women were approached and invited to participate in the study. Those willing to participate gave written informed consent and were interviewed using a pre-tested structured questionnaire administered in Swahili, the local language.

Besides information on demographic characteristics, data were collected on access to sexual and reproductive health services; family planning needs and sexual health status. Women were screened for mild and major depression (using ICD-10 definitions [27]) and for harmful alcohol use with the AUDIT tool [28]. Thereafter study nurses did a clinical examination and collected blood, urine, a cervical swab and a Pap smear. Full details of test procedures and the sample size calculation are provided elsewhere [26]. Urine dipstick detected nitrites and leucocytes. Blood samples were used for malaria microscopy, RPR test for syphilis and serial rapid HIV testing. *Trichomonas vaginalis *and candida were detected in a wet mount, and Nugent's criteria used for diagnosing bacterial vaginosis. Participants were advised to return for test results and, if required, received treatment according to local guidelines. For conditions such as syphilis, malaria and severe anaemia, women with positive results were contacted and asked to return to the clinic immediately. All participants were offered HIV counselling and same-day testing; those testing positive were enrolled in an HIV care and treatment clinic at the study site.

### Study measures and data analysis

Data collected during interviews and laboratory investigations were double entered using Statistical Package for Social Science, version 11.5 (Chicago, USA). For analysis, women were divided into three groups according to the time period since childbirth: early (four weeks to two months), middle (two to six months) and late periods(six to twelve months). These categories were selected to be broadly consistent with the local child immunization schedule and the anticipated patterns of illness in women. Sexually-active women not using contraceptive methods were defined as having an unmet need for contraception. Lactational amenorrhea (exclusive breastfeeding and amenorrhea) was classified as a contraceptive method. Haemoglobin levels below 11 g/l were considered anaemia and a mean corpuscular volume below 81fl microcytic [29].

Intercooled Stata 8.0 (Stata Corporation, College Station, Texas, USA) was used to assess the distribution of sexual behaviour, contraceptive need, morbidity and other variables among women presenting in the early, middle and late periods. Also, the demographic and reproductive health characteristics of these three groups were compared to assess whether systematic differences exist which might be explained by differential health-seeking patterns. For example, we assessed whether poorer or multiparous women were less likely to attend visits in the late period. A chi-square test was used for analysis of binary variables and Mantel-Haenszel odds ratios were calculated. For continuous variables, a Student's *t *test and Wilcoxon's rank-sum test were used for comparing variables with a normal or non-normal distribution respectively. One way analysis of variance was used to compare means of continuous variables in each of the time periods.

## Results

### Characteristics of study population

Of 776 eligible women, 500 (64%) agreed to participate. Reasons given for declining participation included: lack of time (120), need for partner approval before participation (54), and having a child requiring urgent additional care [48]. Most study participants (82%, 395/484) had attended services for well children, such as immunization.

The majority of women were married 431/500 (86%), though only half had entered secondary or tertiary education (Table 1). On average, women were 25.5 years old (standard deviation [sd] = 4.5 years) and 40% were primiparous (195/490). Almost all had attended antenatal care at least once (480/500; 98%), though only 58% of these women had came for four or more antenatal visits (278/480), as recommended by WHO [30]. Four fifths of women (78%; 389/500) had delivered at a health facility. The caesarean section rate was 12% (60/493).

**Table 1:** Demographic and socio-economic characteristics of women accompanying their infant to the child-health clinic at Coast Provincial General Hospital, Mombasa, Kenya

**Variable**	**All women**		**Early period**		**Middle period**		**Late period**		***P***
		**n (%)**		**n (%)**		**n (%)**		**n (%)**	
**Demographics**									
Age mean years (sd) n = 500		25.5 (4.5)		25.7 (4.6)		25.5 (4.7)		25.5 (3.9)	0.82^
**Religion**	N = 499		N = 130		N = 240		N = 129		
Christian		425 (85)		111 (88)		199 (83)		112 (87)	
Muslim		71 (14)		15 (12)		40 (17)		16 (12)	
Other		3 (1)		1 (1)		1 (0)		1 (1)	0.64
**Education level**	N = 500		N = 131		N = 240		N = 129		
Never attended school		17 (3)		1 (1)		8 (3)		8 (6)	
Primary school		249 (50)		65 (50)		122 (51)		62(48)	
Secondary school		191 (38)		47 (36)		93 (39)		51 (40)	
Tertiary education		43 (9)		18 (14)		17 (7)		8 (6)	0.07
**Marital status**	N = 500		N = 131		N = 240		129		
Never married		58 (12)		13 (10)		29 (12)		16 (12)	
Married or cohabitating		431 (86)		117 (89)		203 (85)		111 (86)	
Separated, divorced or widowed		11 (2)		1 (1)		8 (3)		2 (2)	0.47
**Socio-economic**									
**Source of drinking water**	N = 499		N = 131		N = 239		N = 129		
Piped water in house		77 (15)		24 (18)		36 (15)		17 (13)	
Public tap		398 (80)		104 (79)		190 (80)		104 (81)	
Well, borehole or surface water		24 (5)		3 (2)		13 (5)		8 (6)	0.46
**Household income **($/month)	N = 500		N = 131		N = 240		N = 129		
Does not know		90 (18)		27 (21)		46 (19)		14 (11)	
0-41		69 (14)		15 (12)		40 (17)		70 (54)	
41-135		241 (48)		64 (49)		107 (45)		28 (22)	
>135		100 (20)		25 (19)		47 (20)		17 (13)	0.32
**Paternal financial support since childbirth**	N = 497	460 (93)	N = 130	123 (95)	N = 238	217 (91)	N = 129	120 (93)	0.47
**Alcohol use**									
	N = 477		N = 128		N = 227		N = 122		
**Ever drank alcohol**		148 (31)		45 (35)		71 (31)		32 (26)	0.31
**Hazardous or harmful alcohol use|**		11 (2)		3 (2)		6 (3)		2 (2)	0.84
**Drank in pregnancy or breastfeeding**		38 (8)		12 (9)		20 (9)		6 (5)	0.35

Exchange rate of 1$ = 74 Kenyan Shillings; sd = standard deviation; chi-square test unless noted; ^One way analysis of variance.

| In the year preceding pregnancy. Early period: 4 weeks to 2 months after childbirth; Mid period: 2 to 6 months after childbirth; Late period: 6 to 12 months after childbirth.

Participants had given birth a median 3.3 months (IQR = 1.9-6.1) ago. Mixed feeding of infants predominated in each time interval (92%; 454/494) and 96% (124/129) of women were still breastfeeding in the late period. Overall, the demographic characteristics of women in each study period were similar, with the exception of educational status, where women with lower educational levels appeared more likely to attend child-health clinics in the late period.

### Psychosocial vulnerability

Household income was low, with 62% of women below 135$/month (310/500) and 14% less than 41$/month (69/500). Levels of physical and sexual violence are high in this population: more than a third had been coerced into sex (196/497), nearly 10% had been physically forced to have sex (40/491) and 19% had been pushed or hit by an intimate partner (95/499; Table 2). Since childbirth, 8% of women (38/499) had experienced sexual and/or physical violence (4 women reported both forms of violence).

**Table 2:** Sexual behaviour and reproductive health of women in the year after childbirth in Mombasa Kenya

**Variable**	**Early period**	**n (%)**	**Middle period**	**n (%)**	**Late period**	**n (%)**	***P***
**Physical violence**	N = 130		N = 240		N = 129		
Ever pushed or hit by intimate partner		27 (21)		44 (18)		24 (19)	0.84
Pushed or hit since childbirth		8 (6)		12 (5)		10 (8)	0.57
**Sexual violence**							
Ever coerced into having sex	N = 130	48 (37)	N = 238	97 (41)	N = 129	51 (40)	0.77
Ever physically forced to have sex	N = 128	7 (5)	N = 239	28 (12)	N = 126	5 (4)	0.02
Forced to have sex since childbirth	N = 129	0 (0)	N = 239	11 (5)	N = 126	1 (1)	0.009
**Parity**	N = 130		N = 236		N = 124		
1		52 (40)		93 (39)		50 (40)	
2		35 (27)		46 (20)		31 (25)	
3-4		29 (22)		71 (30)		31 (25)	
≥5		14 (11)		26 (11)		12 (10)	0.60
**Infant feeding**	N = 130		N = 235		N = 129		
Exclusive breastfeeding		17 (13)		9 (4)		0 (0)	
Mixed feeding		110 (85)		220 (94)		124 (96)	
Replacement feeding		3 (2)		6 (3)		5 (4)	<0.001
**Fertility plans**	N = 130		N = 240		N = 129		
Wants more children within two years		1 (1)		8 (3)		4 (3)	
Wants more child after two years		64 (49)		124 (52)		79 (61)	
Wants no more children		54 (42)		100 (42)		42 (33)	
Unsure		11 (9)		8 (3)		4 (3)	0.062
**Current contraception**	N = 120		N = 230		N = 125		
Unmet need for contraception		44 (37)		100 (44)		43 (34)	
Modern or traditional contraceptive		16 (13)		80 (35)		68 (54)	
Abstinence since childbirth		60 (50)		50 (22)		14 (11)	<0.001
**Condom use at last sex (sexually- active women)**	N = 70	10 (14)	N = 190	12 (6)	N = 116	6 (5)	0.04^

chi-square test unless noted; ^chi-square test for trend. Early period: 4 weeks to 2 months after childbirth; Middle period: 2 to 6 months after childbirth; Late period: 6 to 12 months after childbirth

Using ICD-10 definitions, 4% of women (18/500) had depression, with 2% of women currently having a major depressive episode (8/499; Table 3). The prevalence of depression diagnosed with DSM-IV criteria was slightly higher (5%; 24/499). Depression increased over the early, middle and late periods with both depression measures (OR = 2.0 per increase in time period, 95%CI = 1.0-3.7; *P *= 0.04 with ICD-10 and OR = 2.1, 95%CI = 1.2-3.8; *P *= 0.008 with DSM-IV). Less than 10% of women in each group reported having drunk alcohol during pregnancy or breastfeeding (38/477), 7 of whom reported having 5 or more drinks on 1 occasion in this time. A relatively small proportion of women used alcohol in a hazardous or harmful manner in the year preceding pregnancy (2%; 11/477).

**Table 3:** Maternal morbidity in the year after childbirth in Mombasa Kenya

**Variable**	**Early period**	**n (%)**	**Mid period**	**n (%)**	**Late period**	**n (%)**	***P***
**General symptoms**							
**Febrile symptoms**	N = 126	8 (6)	N = 237	27 (11)		16/123 (13)	0.17^
**Abdominal pain**	N = 126	21 (17)	N = 237	43 (18)		33/123 (27)	0.03^
**Nutrition, anaemia and malaria**							
**Body mass index**	N = 126		N = 235		N = 126		
Underweight (<18.5 kg/m^2^)		6 (5)		18 (8)		4 (3)	
Normal weight (18.5-25 kg/m^2^)		85 (68)		145 (62)		77 (61)	
Overweight (25-30 kg/m^2^)		25 (20)		59 (25)		30 (24)	
Obese (>30 kg/m^2^)		10 (8)		13 (6)		15 (12)	0.18
**Haemoglobin level**	N = 128		N = 236		N = 125		
Normal		62 (48)		90 (38)		72 (58)	
Normocytic anaemia		27 (21)		45 (19)		24 (19)	
Microcytic anaemia		39 (31)		101 (43)		29 (23)	0.002
**Depression (ICD-10)**	N = 131		N = 239		N = 129		
**Minor depression**		0 (0)		5 (2)		5 (4)	0.03^
**Major depression**		2 (2)		3 (1)		3 (2)	0.61^
**Malaria**	N = 128	3 (2)	N = 236	5 (2)	N = 125	3 (2)	0.98^
**Breast and urinary tract**							
**Breast symptoms**	N = 126		N = 237		N = 123		
Painful, swollen, hot or red breasts		6 (5)		15 (6)		7 (6)	0.92^
Nipple sores or bloody discharge		5 (4)		6 (3)		1 (1)	0.13^
**Breast examination**	N = 125		N = 219		N = 114		
Cracked nipples		4 (3)		5 (2)		1 (1)	0.22
Abscess or mastitis		2 (2)		4 (2)		2 (2)	0.99
**Pain or burning on micturition**	N = 126	13 (10)	N = 237	20 (8)	N = 123	16 (13)	0.28^
**Urinary incontinence**	N = 126	1 (1)	N = 237	4 (2)	N = 123	1 (1)	0.76^
**Urine leukocytes**	N = 121		N = 212		N = 113		
0		75 (62)		132 (62)		90 (80)	
1+		25 (21)		42 (20)		15 (13)	
2+ or 3+		21 (17)		38 (18)		8 (7)	0.014
**Urine nitrites**	N = 127	5 (4)	N = 219	11 (5)	N = 118	2 (2)	0.38^
**Reproductive tract infection and cervical cytology**							
**Symptoms of foul-smelling vaginal discharge**	N = 126	13 (10)	N = 237	33 (14)	N = 123	18 (15)	0.32^
**Abnormal vaginal discharge (examination)**	N = 118	29 (25)	N = 220	53 (24)	N = 108	27 (25)	0.90^
**Bacterial vaginosis**	N = 119	37 (31)	N = 212	78 (37)	N = 116	26 (22)	0.02
**Candida**	N = 121	8 (7)	N = 217	14 (7)	N = 116	14 (12)	0.12^
**Trichomonas vaginalis**	N = 121	9 (7)	N = 217	18 (8)	N = 116	8 (7)	0.88^
**Genital ulcer disease**	N = 118	2 (2)	N = 220	1 (1)	N = 109	4 (4)	0.08^
**Syphilis**	N = 130	0 (0)	N = 237	3 (1)	N = 128	2 (2)	0.33^
**Genital warts**	N = 118	2 (2)	N = 220	5 (2)	N = 109	3 (3)	0.63^
**Cervical cytology Ω**	N = 97		N = 195		N = 103		
Normal		76 (78)		178 (91)		100 (97)	
Atypical squamous cells of undetermined significance		8 (8)		9 (5)		1 (1)	
Low-grade squamous intraepithelial lesion		9 (9)		5 (3)		1 (1)	
High-grade squamous intraepithelial lesion		4 (4)		3 (2)		1 (1)	0.002

Ω 42 Pap smear slides were of unsatisfactory quality. chi-square test unless otherwise indicated; ^chi-square test for trend. Early period: 4 weeks to 2 months after childbirth; Middle period: 2 to 6 months after childbirth; Late period: 6 to 12 months after childbirth

### Sexual behaviour and contraceptive use

Sexual activity increased with duration of time since childbirth, from 54% of women in the early period (71/131) to 80% (193/240) in the middle and 90% (116/129) in the last interval (*P *< 0.001). The large majority (86%, 296/344) of sexually-active women had not used condoms since childbirth (Table 2).

Though less than half said their most recent pregnancy was planned (46%; 231/500), in all study periods around a third of women had an unmet need for contraception (38% overall; 187/497). Of women using contraception, injectable methods were most commonly used (114 women), followed by oral contraception [19] and only three women were using an intra-uterine device.

Levels of contraception were incongruous with women's stated fertility intentions - only 3% of women desired more children within two years (13/499). Half in the early period expressed a desire for more children (50%; 65/130), while two thirds of women in the last group mentioned this (64%, 83/129; *P *= 0.02).

### Self-reported health problems and health seeking behaviour

Only 13% of participants who had delivered in a health facility said they received information about warning signs of postpartum complications (48/376), and 23% were told to return for a postpartum check-up (87/381). About 10% of women had attended the check-up visit (58/500). More than a third had experienced health problems since childbirth (178/302; 37%), of whom 43% sought health care (71/164). At time of interview, 53% of women (257/483) said they felt unwell. The most frequent symptoms were abdominal pain, fever and foul-smelling vaginal discharge, all of which increased with duration of time since childbirth. Abdominal pain and vaginal discharge were strongly associated: women with pain were 4.3 times more likely to report vaginal discharge than other women (95%CI = 2.4-7.7; *P *< 0.001).

The vast majority believed they would benefit from a health worker visit to their home in the postpartum period (462/492; 94%). As an indicator of level of access to sexual and reproductive health services in the area, only 6% had previously been screened for cervical cancer (27/485).

### Clinical morbidity

About one in twenty women was underweight (6%; 28/487), similar to the proportion obese (8%; 38/487). The mean haemoglobin was 10.5 g/l (sd = 1.8 g/l), with more than half the population anaemic (54%; 265/489) and 5% having severe anaemia (hb < 7 g/L; 26/489). Anaemia was less common in women who reported taking iron supplementation as prescribed during pregnancy (50%; 128/254) than women who took some iron (56%; 81/144), or did not take any supplementation (62%; 56/91; *P *= 0.05, chi-square test for trend). Higher levels of anaemia were also seen in underweight women (76%; 19/25), women who had a caesarean section (75%; 44/59) and those with an income below 41$/month (59%; 40/68). Few women had malaria parasitaemia, consistent with reported levels of bed net use - about two thirds reported they slept under a net the previous night (68%; 339/497).

Leukocyturia and positive nitrite status were most frequent in the early and middle periods. Reproductive tract infections were common, with an overall prevalence of bacterial vaginosis of 32% (141/447) and candida infection of 8% (36/454). Unlike these latter conditions, *Trichomonas vaginalis *was associated with genital symptoms. Of women reporting vaginal discharge symptoms, 14% had trichomoniasis (8/57), while 7% of women without discharge symptoms had trichomoniasis (27/393; *P *= 0.059). Compared with women in the early and mid period, a higher proportion in the late group had genital ulcers (4%; 4/109) or warts (3%, 3/109) and syphilis (2%, 2/128). Three quarters of participants reported having accessed HIV testing during pregnancy or childbirth (372/496) and 54/496 (11%) were HIV infected. Abnormal Pap smears, more common in the period closest to childbirth, identified a total of 4% (15/395) low-grade squamous intraepithelial lesions and 2% (8/395) high-grade squamous intraepithelial lesions.

## Discussion

The study describes the burden and patterns of morbidity in the year after childbirth. Conditions associated with pregnancy, childbirth or breastfeeding predominate in the early and middle periods, while illnesses stemming from a return of sexual function and a transition to reproductive potential were commoner in the latter period. Broadly speaking, therefore, in the initial period after childbirth, interventions targeting breast health, the urinary tract and anaemia are most pertinent, while promotion of sexual health and prevention of unintended pregnancy have more importance in subsequent periods. It is, however, difficult to make a definitive distinction between needs of women in each time period and whether morbidities in these periods are related to pregnancy, return of reproductive potential or intercurrent illness. For example, throughout the first year, high levels of fever, abdominal pain and abnormal vaginal discharge make vigilance for sepsis important, a major cause of maternal mortality in Africa [16].

Over one third reported health problems and overall perceived health status was low. Among women in the early postpartum in Zambia, 84% reported at least one health problem [31]. A large study in India found 23% had health problems in the early postpartum [32] with even higher levels reported in Sri Lanka [33]. Taken together, it is thus clear that health problems are common among women after childbirth, but health care services are not commiserate with these levels of ill health.

Prevalence of treatable reproductive tract infections was high, similar to previous findings in Kenya [34]. The association between bacterial vaginosis and postpartum endometritis is potentially important in this population, but also its links with complications in a future pregnancy such as preterm delivery and preterm rupture of membranes [35-39]. STIs generally increased with each period since childbirth, concurring with the reported increase in sexual activity over time and the low condom use throughout. Low condom use, comparable to levels in a similar population in Zimbabwe [9], may be due to women perceiving themselves at low risk for pregnancy in the postpartum [40]. Absence of syphilis infection within two months of childbirth likely reflects the high coverage of antenatal care and quality of these services. As reported previously [25], acceptability of HIV testing was high among study participants and around four fifths received their results. Women with HIV infection have high morbidity and mortality in the year after childbirth [11] - for example, in our population, two thirds had anaemia and 20% reported current febrile symptoms [26]. These women require specific HIV care and treatment in addition to the needs of women described in this paper. Among the whole study population, levels of violence were remarkably high, though comparable with previous prevalence estimates in the general population in eastern Africa [41].

More than half of recent pregnancies were unplanned, and despite a desire to postpone pregnancy, more than a third of women had unmet need for contraception. Adequate birth spacing confers many benefits for women and their children and WHO recommends at least two to three years between pregnancies [42]. Women attending child health clinics need to be made aware of the benefits of adequate birth spacing, supported in their fertility choices, and to be offered contraception and dual protection. With adequate family planning services, uptake of hormonal contraception is high among postpartum women in Kenya [10], as much as 72% in a study [8].

Over half the women, and three quarters of some high-risk groups (such as those who had a caesarean section), had anaemia. Anaemia may be exacerbated by pregnancy and childbirth, and current WHO recommendations for populations with a severe prevalence of anaemia (>40%, as in this population) are that preventative and therapeutic iron supplementation should be given to all women of childbearing age and routinely in the postpartum [43]. Giving women attending child health clinics haematinic supplements, which are highly effective in reducing anaemia [44], may prevent anaemia in subsequent pregnancies.

That demographic characteristics of women in each of the three time periods were similar, suggests that health-seeking patterns were not strongly influenced by socio-demographic characteristics. These characteristics were, however, important in a study in rural Zambia [45]. In our population, differences between the study groups in experiences of sexual violence and depression indicates that psychosocial experiences may affect the timing and number of visits to child health centres.

However, perhaps women with severe postpartum depression in early period were less likely to bring their child for immunizations, spuriously lowering depression in this group. There is a biological explanation for higher rates of abnormal Pap smears shortly after childbirth. False positive smears can be caused by cervical inflammation related to the birth process, which regresses gradually over time [46,47]. Overall, coverage of cervical cancer screening in Kenya remains notably low and this cancer the most common malignancy among women in the country [48].

This study has several limitations. While factors such as sexual activity, rate of caesarean section and prevalence of HIV are broadly comparable to those in urban areas of Kenya [49,50], it is difficult to generalise the findings to rural areas, or to lower-level health facilities. In addition, women who do not attend child-health clinics, women whose child has died, or the substantial proportion who declined study participation may differ in important ways from the study population. Rates of morbidity are also likely to vary markedly between populations, in for example postpartum clinics and child-health clinics, or between community and hospital-based samples. Moreover, self-reported symptoms and behaviour incur recall and social desirability bias, though can be useful indicators of perceived health status.

Evaluation of morbidity in the whole year since childbirth makes it difficult to compare our findings with studies that included only women in the conventional six-week postpartum. However, including a longer period is a strength of this study, as it acknowledges the high risk women have for mortality between six weeks and a year after childbirth [14,15]; is cognisant of the need for a continuum of care throughout this time; and presents evidence of the potential benefits of optimising the interface between women and health workers during child health visits to address the substantial burden of disease women have throughout this period.

## Conclusion

Although most of the common problems women experienced were not life-threatening, they likely have marked influence on their wellbeing and health status in the long run. Most conditions could be addressed through provision of health promotion and preventive interventions, which is highly complementary to the intrapartum focus on acute severe problems.

The year after childbirth, an important part of women's lifecycle, is a neglected component of the continuum of care and support for women. Women in this period require interventions such as prevention and treatment of anaemia and reproductive tract infections, but also provision of family planning counselling and services, mental health services and distribution of condoms. Though many indicated they might benefit from home visits, this intervention requires additional study [51,52]. Interface with women at child-health clinics, however, offers an opportunity to provide or facilitate entry to sexual and reproductive health services, and to encourage their long-term use.

The potential benefits of extending postpartum services beyond the conventional six-week period after childbirth may necessitate revision of the classic approach to postpartum care. The findings of this study indicate that consideration is warranted of recommending an early visit around four to six weeks postpartum and later visits at three, and six to nine months after childbirth, which concurs with the immunization schedule in many countries. Adequate care for women in the year after childbirth, possibly provided in child-health centres, could make an important contribution to international commitments to improve maternal health [53].

## Competing interests

The authors declare that they have no competing interests.

## Authors' contributions

MFC participated in the design of the study and drafted the manuscript. SL and NK participated in the design of the study and development of the initial draft. EY, CN and MO participated in the study coordination, collection of data and helped to draft the manuscript. MT made a substantial contribution to the study design, analysis and interpretation of findings. All authors read and approved the final manuscript.

## Pre-publication history

The pre-publication history for this paper can be accessed here:


